# Vitamin D Deficiency as an Independent Predictor for Plaque Vulnerability and All-Cause Mortality in Patients with High-Grade Carotid Disease

**DOI:** 10.3390/jcm14145163

**Published:** 2025-07-21

**Authors:** Stephanie Kampf, Olesya Harkot, Rodrig Marculescu, Svitlana Demyanets, Markus Klinger, Wolf Eilenberg, Johann Wojta, Christoph Neumayer, Stefan Stojkovic

**Affiliations:** 1 Department of Surgery, Division of Vascular Surgery, Medical University of Vienna, 1090 Vienna, Austria; stephanie.kampf@meduniwien.ac.at (S.K.); markus.kliniger@meduniwien.ac.at (M.K.); wolf.eilenberg@meduniwien.ac.at (W.E.); 2 Department of Internal Medicine II, Division of Cardiology, Medical University of Vienna, 1090 Vienna, Austria; olesya.harkot@meduniwien.ac.at (O.H.); stefan.stojkovic@meduniwien.ac.at (S.S.); 3 Department of Laboratory Medicine, Medical University of Vienna, 1090 Vienna, Austria; rodrig.marculescu@meduniwien.ac.at (R.M.); svitlana.demyanets@meduniwien.ac.at (S.D.); 4 Department of Laboratory Medicine, Clinic Hietzing, 1100 Vienna, Austria; 5 Core Facilities, Medical University of Vienna, 1090 Vienna, Austria; johann.wojta@meduniwien.ac.at

**Keywords:** morbidity, mortality, plaque morphology, chemiluminescence immunoassay, AHA classification

## Abstract

**Objectives:** The mechanisms linking vitamin D deficiency to carotid artery stenosis (CAS) remain unclear. Data on cardiovascular outcomes in CAS patients with vitamin D deficiency are limited. We investigated the association of vitamin D deficiency with carotid plaque morphology and patient outcomes in high-grade CAS. **Methods:** A total of 332 patients undergoing carotid endarterectomy for symptomatic (n = 113, 34%) or asymptomatic (n = 219, 66%) CAS were included. Preoperative vitamin D levels were measured, and duplex sonography was used to assess luminal narrowing. Associations of vitamin D with clinical presentation were analyzed using univariate and multivariate linear regression. For vitamin D deficiency and the prediction of major adverse cardiovascular events (MACE) and all-cause mortality, the Cox proportional hazard regression model was used. **Results:** The median age was 69 years (interquartile range (IQR) 64–74), and 94 (29.3%) patients were female. Vitamin D deficiency was present in 84 (25%) patients. Symptomatic patients had significantly lower vitamin D levels (41.2 nmol/L, IQR 25.1–63.5) than asymptomatic patients (51.6 nmol/L, IQR 30.5–74.3, *p* = 0.011). Patients with echolucent (44.9 nmol/L, IQR 27.4–73.7) or mixed plaques (39.2 nmol/L, IQR 22.9–63.5) had lower vitamin D levels than those with echogenic plaques (52.3 nmol/L, IQR 34.1–75.7). Vitamin D deficiency predicted MACE and all-cause mortality with an adjusted HR of 1.6, 95% CI of 1.1–2.6, and *p* = 0.030 and an HR of 2.2, 95% CI of 1.3–3.6, and *p* = 0.002, respectively, in a multivariable Cox proportional hazard regression model. **Conclusions:** A deficiency in vitamin D was correlated with unstable plaque characteristics and symptomatic CAS. Furthermore, vitamin D deficiency was associated with long-term adverse cardiovascular outcomes and mortality, suggesting its potential as a modifiable risk factor for improved risk stratification in patients undergoing carotid endarterectomy.

## 1. Introduction

Atherosclerosis, marked by chronic inflammation within the vascular wall, facilitates plaque formation in carotid arteries. Carotid artery stenosis (CAS) accounts for up to 20% of ischemic strokes globally, making it a critical target for prevention strategies [1,2]. Especially in asymptomatic CAS, stroke prevention is challenging. The current management for symptomatic and asymptomatic CAS of “average” risk includes best medical therapy, carotid endarterectomy, or carotid artery stenting. Currently, the most important conservative measures are lifestyle changes, antiplatelet therapy, lipid-lowering therapy, and control of blood pressure [3]. Dietary antioxidants and supplements have been discussed, but up to now, supporting evidence is limited [4].

Vitamin D contributes significantly to physiological equilibrium and is essential for cardiovascular health [5]. Inadequate dietary intake or sun exposure are the main causes of diminished serum levels of vitamin D. Low levels of vitamin D have been linked to traditional cardiovascular risk factors such as hypertension, dyslipidemia, insulin resistance, and obesity, but results from interventional trials of supplementation remain inconsistent [6,7,8,9]. Vitamin D deficiency has also been reported to be associated with higher carotid intima–media thickness (CIMT) and more prominent atherosclerotic plaques in smokers [10]. Data on the association between vitamin D deficiency and carotid plaque morphology, especially in patients with high-grade CAS, are limited.

There is still insufficient data on cardiovascular outcomes in patients with CAS suffering from vitamin D deficiency. Vitamin D insufficiency could be regarded as a new, readily modifiable risk factor among affected individuals with high-grade CAS. Therefore, the current study aimed to investigate whether vitamin D deficiency is associated vulnerable carotid plaque characteristics and adverse long-term cardiovascular outcomes in patients undergoing carotid endarterectomy.

## 2. Patient and Methods

### 2.1. Patient Population

In the present prospective, single-center, observational cohort study, a total of 332 consecutive patients from 2012 to 2018 with CAS undergoing carotid endarterectomy were included. All surgical procedures were performed at the Department of Vascular Surgery. This single-center study was approved by the local institutional review board and was performed in accordance with the principles of the Declaration of Helsinki. All patients gave written informed consent. The study was listed at clinicaltrials.gov (NCT06473337). All patients had to be >18 years and have an indication for carotid endarterectomy to be suitable for the study. The indication for surgery included symptomatic CAS ( ≥50%) or high-grade asymptomatic CAS ( ≥70%) [3]. Patients who underwent previous carotid interventions, missed preoperative vitamin D measurements, or had incomplete follow-up data were excluded. Furthermore, patients with hemorrhagic, lacunar, or cardioembolic stroke were excluded from the study. Symptomatic patients had a stroke or transient ischemic attack ipsilateral to the CAS within 6 months prior to surgery.

All surgeries were performed by trained vascular surgeons. The degree of CAS was assessed by carotid Duplex sonography and/or computed tomography angiography, as previously described [11]. Duplex sonography examinations were performed by independent, trained medical technical assistants, performing on average 10 scans per day.

Patient follow-ups were performed at 3 months and 1, 3, 5, and 10 years for up to 10 years after the surgery. At each follow-up clinical evaluation, symptom status, medical history, carotid duplex ultrasound, and assessment of primary and secondary endpoints were performed. Only 3 patients were lost to follow-up and were excluded from further analysis, and 99.9% of the patients completed the follow-up. The primary cardiovascular endpoint was defined as the composite of cardiovascular death, myocardial infarction, TIA, or stroke, as well as atherosclerosis progression in the coronary or peripheral arteries requiring either interventional (percutaneous coronary intervention or peripheral balloon angioplasty with and without stenting) or surgical revascularization (aortocoronary bypass or peripheral bypass), summarized as MACE [12]. The secondary endpoint was defined as all-cause mortality. A flow chart of the study is shown in Supplemental Figure S1.

### 2.2. Carotid Plaque Morphology

The preoperative morphological evaluation of CAS was based on carotid Duplex ultrasound and/or computed tomography angiography, as previously described [13,14,15]. Plaques were classified according to ultrasound echogenicity into echolucent (non-calcified, unstable, or vulnerable plaque), echogenic (calcified, stable, or non-vulnerable plaque), and mixed plaques (partly calcified) [16] by the clinic’s expert vascular laboratory.

For histological classification, plaques were formalin-fixed, embedded in paraffin, and stained with hematoxylin–eosin and elastic van Giessen staining, according to standard in-house protocol, and classified according to a modified American Heart Association (AHA) classification based on a morphological description, as recently described [12]. Vulnerable plaques were defined as grade VI (complex lesions with possible surface defect) according to AHA classification [17,18]. Non-vulnerable plaques were defined as grades IV (atheroma), V (fibroatheroma), VII (calcified plaques), and VIII (fibrotic plaque without lipid core) [17,19].

## 3. Vitamin D and Other Assessed Laboratory Parameters

Baseline vitamin D levels were measured prior to surgery in all patients using blood serum samples. Patients with preoperative supplementation of vitamin D were excluded. All laboratory analyses were performed at the Department of Laboratory Medicine, according to ISO 9001 [20] and ISO 15,189 [21] quality standards. Serum 25-hydroxy-vitamin D was measured by the Liaison Total 25-Hydroxy-Vitamin D chemiluminescence immunoassay (CLIA) on Liaison XL immunology analyzers (DiaSorin, Saluggia, Italy). Values were given as nanomole per L (nmol/L), and vitamin D levels below 30 nmol/L were considered a deficiency [22].

Total cholesterol, low-density lipoproteins (LDL), high-density lipoproteins (HDL), triglycerides, lipoprotein (a) (Lp(a)), and high-sensitivity C-reactive protein (hs-CRP) were measured on a Cobas e602 immunochemistry module (Roche, Basel, Switzerland)) according to the instructions of the manufacturer.

## 4. Statistical Analysis

Categorical variables were summarized as counts and percentages and were compared by the χ^2^ test or the Fisher’s exact test as appropriate. Continuous variables were expressed as median and interquartile range (IQR) and compared using the t-test and one-way ANOVA. The Mann–Whitney U test was used in case of non-normal distribution. Sample size calculation analysis showed that in a cohort with an event rate of 25%, given a power of 0.8 and a significance level of 0.01, we required 200 patients to detect a difference of 50% in vitamin D serum levels between patients with and without MACE during the follow-up. Univariate and multivariate linear regression models were fit to evaluate the associations of vitamin D with baseline clinical presentation (symptomatic/asymptomatic). The Cox proportional hazard regression model was fit to assess whether vitamin D deficiency could significantly predict long-term cardiovascular outcomes and all-cause mortality. In order to investigate whether vitamin D independently predicts MACE and all-cause mortality, a multivariable Cox proportional hazard regression model was fit and adjusted for age, sex, and most important clinical risk factors: hypertension, diabetes, coronary and peripheral artery disease, smoking, hsCRP, and LDL. The variables for the multivariable model were predefined at the beginning of the study, based on literature, as the most prevalent risk factors in patients with carotid artery stenosis [3,23,24], as well as the variables which were significantly associated with the primary endpoint in univariate Cox regression analysis. Kaplan–Meier survival plots were constructed in groups according to vitamin D deficiency to compare the time-dependent discriminative power of vitamin D levels. As the median follow-up time of the study was 5.7 years and the standard error thereafter was above 10%, six years of the follow-up were reported in the Kaplan–Meier plots. Two-sided *p*-values of 0.05 indicated statistical significance. SPSS 22.0 (IBM Corporation, Armonk, NY, USA) and STATA version 12 (Stata Corp LLC, College Station, TX, USA) were used for all statistical analyses.

## 5. Results

Baseline clinical characteristics of the study cohort are shown in Table 1. The median age was 69 years (IQR 64–74), 238 patients (71.7%) were male, and 113 (34%) patients presented with symptomatic CAS. The median vitamin D level in the overall patient population was 47.3 nmol/L (IQR 29.2–71.0). Vitamin D deficiency, i.e., vitamin D levels < 30 nmol/L, was observed in 84 (25%) patients. Patients with vitamin D deficiency were more often diabetic (45.2% vs. 29.4%, *p* = 0.011), had chronic obstructive pulmonary disease (COPD) (25% vs. 13.7%, *p* = 0.014), and were active smokers (39.3% vs. 24.6%) with significantly more pack-years (Table 1).

Furthermore, patients with vitamin D deficiency had lower HDL (41 mg/dL, IQR 30–54 vs. 50 mg/dL, IQE 40–62, *p* < 0.001) and higher hs-CRP levels (0.37 mg/dL, IQR 0.19–0.98 vs. 0.28 mg/dL IQE 0.13–0.67, *p* = 0.045) at baseline, as compared to patients without vitamin D deficiency. Just over one third of the patients presented with symptomatic CAS (34%), and the median grade of stenosis was 90% (Table 2).

**Figure 1:**
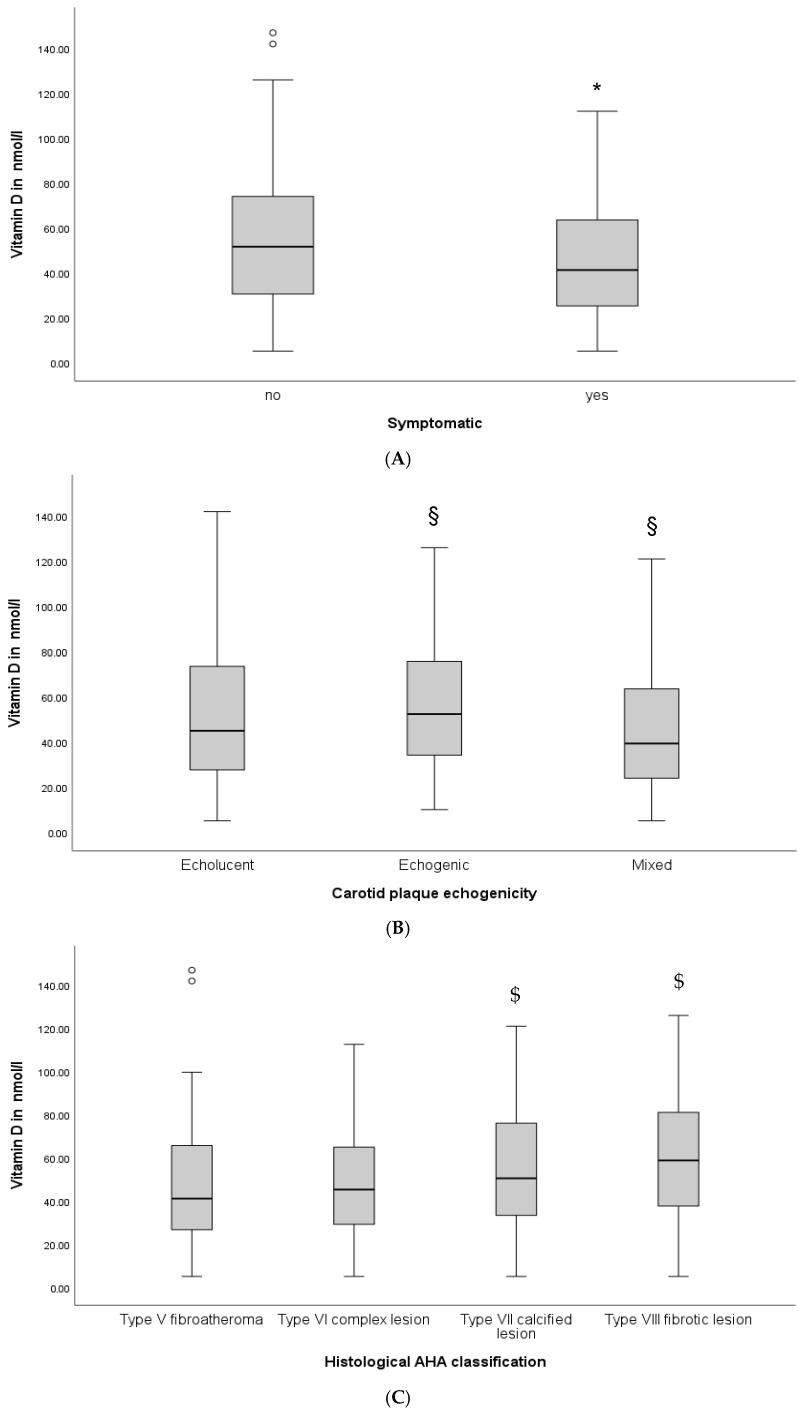
Vitamin D levels according to clinical symptom presentation (**A**), carotid plaque morphology (**B**), and histological AHA classification (**C**). Baseline vitamin D levels, clinical presentation, and carotid plaque morphology were determined as described in the Section 2. (**A**) depicts vitamin D levels in patients with symptomatic and asymptomatic carotid artery stenosis. (**B**) depicts vitamin D levels according to the carotid ultrasound and (**C**), according to the AHA histological classification. Concentrations were depicted as median with interquartile range and given in nmol/L. *p*-values < 0.05 were considered significant. * *p* < 0.05, as compared to asymptomatic; § *p* < 0.05, as compared to echogenic; and $ *p* < 0.05, as compared to type V fibroatheroma and type VI complex lesion, respectively.

Symptomatic patients had significantly lower vitamin D levels (41.2 nmol/L, IQR 25.1–63.5), as compared to asymptomatic patients (51.6 nmol/L, IQR 30.5–74.3, *p* = 0.011, Figure 1A). Patients with echolucent (44.9 nmol/L, IQR 27.4–73.7) or mixed plaques (39.2 nmol/L, IQR 22.9–63.5) in the preoperative carotid ultrasound had lower vitamin D levels, as compared to patients with echogenic plaques (52.3 nmol/L, IQR 34.1–75.7, ANOVA for between-group comparisons; *p* = 0.013, Figure 1B). Furthermore, we observed differences in vitamin D levels between groups, according to the histological AHA classification of carotid plaques. Patients with type VII calcified and type VIII fibrotic lesions had significantly higher vitamin D levels, as compared to patients with type V fibroatheroma and type VI complex lesions (Figure 1C, ANOVA for between-group comparisons; *p* = 0.012). Finally, vitamin D deficiency was independently associated with symptomatic carotid plaques (OR 2.1, 95% CI 1.1–3.9, *p* = 0.021) in the multivariable regression model after adjustment for age, sex, diabetes, coronary and peripheral artery disease, smoking, hsCRP, and LDL. Besides vitamin D deficiency, the presence of coronary and peripheral artery disease and LDL levels were significantly associated with symptomatic carotid artery stenosis in the multivariable regression model.

**Figure 2:**
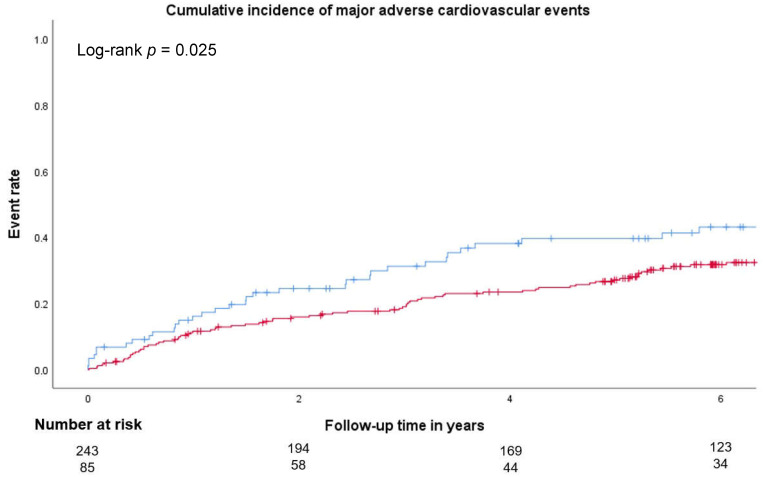
Cumulative incidence of major adverse cardiovascular events in patients with and without vitamin D deficiency. Kaplan–Meier analyses showed the cumulative incidence of major adverse cardiovascular events (primary endpoint) in patients with (blue line) and without (red line) vitamin D deficiency at baseline. Vitamin D levels were assessed as described in the Section 2. *p*-values < 0.05 were considered significant.

After a median follow-up time of 5.7 years (IQR 2.9–7.3), 117 (35.2%) patients had MACE. MACE occurred significantly more often in patients with vitamin D deficiency prior to carotid endarterectomy (Figure 2, log-rank *p* = 0.025). Both symptomatic (log-rank *p* = 0.030) and asymptomatic (log-rank *p* = 0.028) patients with vitamin D deficiency had significantly more often MACE during the follow-up (Supplemental Figure S2).

Baseline vitamin D deficiency predicted MACE with an HR of 1.5, 95% CI of 1.1–2.2, and *p* = 0.026 in univariate Cox proportional hazard regression analysis. A multivariable model was fit to investigate whether vitamin D deficiency independently predicted the MACE (Table 3) and was adjusted for age, sex, hypertension, diabetes, coronary and peripheral artery disease, smoking, hsCRP, and LDL. Vitamin D deficiency independently predicted MACE with an adjusted HR of 1.6, 95% CI of 1.1–2.6, and *p* = 0.030 (Table 3). Besides vitamin D deficiency, only peripheral artery disease was significantly associated with MACE in the multivariable model (HR 2.4, 95% CI 1.4–3.9, *p* < 0.001).

**Figure 3:**
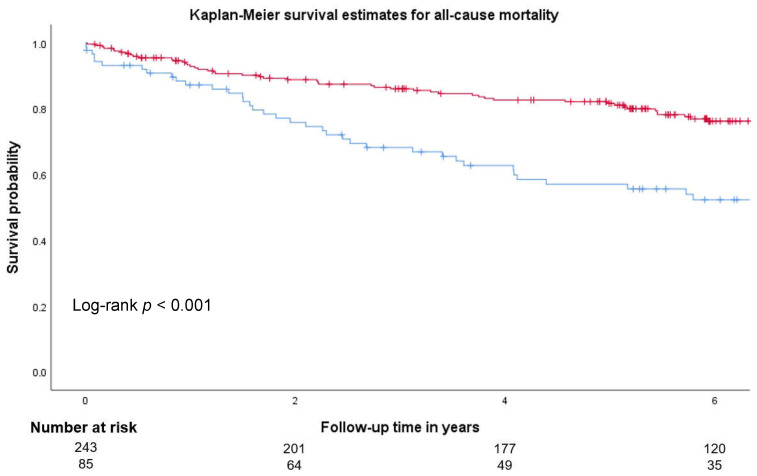
Kaplan–Meier survival curve in patients with and without vitamin D deficiency. Kaplan–Meier curves illustrated overall mortality rates in patients with (blue line) and without (red line) vitamin D deficiency at baseline. Vitamin D levels were assessed as described in the Section 2. *p*-values < 0.05 were considered significant.

Finally, patients with vitamin D deficiency undergoing carotid endarterectomy had significantly higher all-cause mortality during the median follow-up time of 5.7 years in the Kaplan–Meier survival analysis (Figure 3, log-rank *p* < 0.001). Again, this was true independently of the baseline symptom presentation, as both symptomatic (log-rank *p* = 0.013) and asymptomatic (log-rank *p* = 0.006) patients with vitamin D deficiency had significantly higher all-cause mortality, as compared to patients without vitamin D deficiency (Supplemental Figure S3).

Furthermore, in univariate Cox proportional hazard regression analysis, vitamin D deficiency predicted long-term all-cause mortality in this group of patients (HR 2.2, 95% CI 1.5–3.3, *p* < 0.001, Table 3). In multivariable Cox proportional hazard regression analysis, vitamin D deficiency independently predicted all-cause mortality with an adjusted HR of 2.2, 95% CI of 1.3–3.6, and *p* = 0.002, after adjustment for age, sex, hypertension, diabetes, coronary and peripheral artery disease, smoking, hsCRP, and LDL (Table 3). In addition to vitamin D deficiency, age and hsCRP independently predicted all-cause mortality in the multivariable model (HR 1.05, 95% CI 1.02–1.08, and *p* < 0.001 for age and HR 1.2, 95% CI 1.1–1.3, and *p* < 0.001 for hsCRP, respectively).

## 6. Discussion

Studies investigating vitamin D deficiency in patients with CAS are limited and have mainly focused on its association with subclinical carotid atherosclerosis [25,26,27,28,29]. In our study, we could show for the first time that patients with vitamin D deficiency more frequently exhibited signs of vulnerable carotid plaques in both preoperative duplex sonography and postoperative histological analysis. This translated into an independent association of vitamin D deficiency with symptomatic carotid artery stenosis. Furthermore, vitamin D deficiency was identified as a potential independent and modifiable risk factor for MACE and all-cause mortality during long-term follow-up after carotid endarterectomy. While the Tromsø study found an association only in non-smokers [26], the Northern Manhattan study reported a direct link between low vitamin D levels and increased carotid intima–media thickness (CIMT) and plaque thickness [27].

Additionally, more recent studies reported an inverse association between circulating 25-hydroxy-vitamin D and CIMT [30,31]. Intima–media thickness and epicardial fat thickness were reported to be increased in patients with vitamin D deficiency [31]. In our cohort, patients with vitamin D deficiency more often presented with echolucent or mixed plaques, which are known indicators of plaque instability and higher stroke risk. [32]. Notably, vitamin D deficiency predicted MACE and mortality independently of smoking status and diabetes.

Emerging evidence suggests a potential link between vitamin D deficiency and early atherosclerosis, independent of traditional risk factors [25,33,34]. In a cross-sectional study, low vitamin D levels were associated with magnetic resonance imaging-detected intraplaque hemorrhage in 65 patients with CAS [35]. Up until now, our study was the first to demonstrate an association of vitamin D deficiency with high-risk histological vulnerable carotid plaque formation in a large cohort of symptomatic and asymptomatic CAS patients. Vitamin D is suspected to be involved in the modulation of smooth muscle cell proliferation, inflammation, and thrombosis [8]. Since plaque vulnerability is linked to vascular inflammation [36], this supports our hypothesis that vitamin D deficiency plays a crucial role in high-risk plaque formation in CAS. In line with this, circulating vitamin D levels were independently associated with symptomatic carotid stenosis, even after adjustment for clinical risk factors. In addition, vitamin D deficiency was present in 25% of our cohort, highlighting it as a common and potentially modifiable risk factor prior to carotid endarterectomy.

Vitamin D deficiency was shown to be associated with increased mortality after myocardial infarction [37]. In our study, symptomatic and high-grade asymptomatic CAS patients with vitamin D deficiency showed a 2.5-fold higher all-cause mortality and increased MACE during follow-up. CAS is known as a marker of systemic atherosclerotic burden, which was reflected in our cohort, where 33.1% also had peripheral artery disease and 37.6% coronary artery disease [38,39]. Low vitamin D levels may be a consequence of the arteriosclerotic disease-related lifestyle. Reduced physical activity and therefore lower UV-induced vitamin D production may act as potential confounders. However, vitamin D deficiency remained a significant predictor for mortality and MACE after multivariable adjustments, including coronary and peripheral artery disease, and diabetes, as well as LDL and hsCRP.

## 7. Limitations

A number of limitations require discussion. One of our major limitations was the limited number of cases with vitamin D deficiency due to single-center conduction. Therefore, larger, multicenter studies would be needed. However, the high event rate of 35% and long-term follow-up of up to 10 years should overcome statistical concerns. Additionally, each of our patients had only one measurement of vitamin D levels, and vitamin D status was not adjusted to seasonal differences due to UV exposure. However, as patient recruitment and blood sampling were evenly distributed throughout the study period (2012–2018), seasonal variation in vitamin D levels was likely mitigated. Parathormone, to rule out a possible influence of a secondary hyperparathyroidism, was not routinely measured, albeit none of the patients had terminal renal failure. The observational design of our study also excluded the mechanistic insight of the impact of vitamin D on carotid plaque morphology. It is possible that the observed association between low vitamin D levels and carotid plaque vulnerability reflects a broader phenotype of poor health rather than a direct causal relationship. Individuals with frailty, chronic systemic inflammation, or limited mobility may have reduced sun exposure and dietary intake, leading to lower vitamin D levels. This phenomenon could explain why observational studies consistently reported strong associations between vitamin D deficiency and cardiovascular risk, while interventional trials of supplementation yielded mixed results [40]. Measurement of vitamin D can be easily performed, and future prospective studies are needed to further recommend a supplementation therapy in patients with high-grade CAS.

## 8. Conclusions

Vitamin D levels are associated with high-risk atherosclerotic plaque morphology and clinical presentation in patients with high-grade carotid artery stenosis. Patients with vitamin D deficiency more often have vulnerable and symptomatic carotid atherosclerotic plaques. Finally, vitamin D deficiency is independently associated with long-term adverse cardiovascular outcomes and all-cause mortality. Thus, vitamin D deficiency might represent a novel and easily modifiable risk factor for long-term outcomes in patients undergoing carotid endarterectomy.

## Figures and Tables

**Table 1:** Clinical characteristics of the patient cohort.

	Overall (n = 332)	Vitamin D Deficiency (n = 84)	No Vitamin D Deficiency (n = 248)	*p*-Value
**Demographics**				
Age	69 (±8.7)	68 (±7.6)	70 (±9.2)	0.193
Sex (male)	238 (71.7)	61 (72.6)	177 (71.7)	0.889
**Comorbidities and risk factors**				
History of stroke/TIA	56 (21.7)	13 (21.3)	43 (21.8)	1.000
Acute myocardial infarction	66 (19.9)	19 (22.6)	47 (18.9)	0.529
Coronary artery disease	125 (37.6)	27 (32.1)	98 (39.5)	0.242
Peripheral artery disease	110 (33.1)	26 (30.9)	84 (33.9)	0.688
Arterial hypertension	298 (89.7)	77 (91.6)	221 (89.1)	0.676
Hypercholesterolemia	230 (69.3)	60 (71.4)	170 (68.5)	0.784
Diabetes mellitus type 2	111 (33.4)	38 (45.2)	73 (29.4)	0.011
Obesity (BMI > 30 kg/m^2^)	75 (22.5)	24 (28:5)	51 (20.5)	0.131
Smoking active	94 (28.3)	33 (39.3)	61 (24.6)	0.035
COPD	52 (15.7)	21 (25.0)	31 (13.7)	0.014
**Concomitant medication**				
Statin	312 (93.9)	79 (94)	233 (93.9)	0.776
Antiplatelet	309 (93.1)	80 (95.2)	229 (92.3)	0.792
Betablocker	180 (54.2)	48 (57.1)	132 (53.2)	0.610
ACE inhibitor/ARB	241 (72.6)	65 (77.3)	176 (70.9)	0.222
Insulin	24 (7.2)	9 (10.7)	15 (5.2)	0.178
Metformin	105 (32.5)	38 (45.2)	67 (25.9)	0.081

Continuous data are shown as the median (interquartile range), age is shown as mean with standard deviation. Dichotomous data are shown as n (%). The Mann–Whitney test was used for the statistical comparison of continuous variables and Fisher’s exact test for the categorical variables. TIA, transitory ischemic attack; BMI, body mass index; ACE, angiotensin-converting enzyme; ARB, angiotensin receptor blocker.

**Table 2:** Clinical and morphological characteristics of carotid artery stenosis.

	Overall (n = 332)	Vitamin D Deficiency (n = 84)	No Vitamin D Deficiency (n = 248)	*p*-Value
**Characteristics of carotid artery stenosis**				
Symptomatic	113 (34)	34 (40.5)	79 (31.9)	0.096
Grade of stenosis	90 (80–95)	90 (80–95)	90 (80–95)	0.654
US plaque morphology				
Echogenic	114 (34.3)	22 (26.1)	92 (37.1)	0.094
Mixed	46 (13.8)	16 (19.0)	30 (12.1)	
Echolucent	145 (43.6)	40 (47.6)	105 (42.3)	
Histological AHA classification				
Type V fibroatheroma	73 (21.9)	22 (26.12)	51 (20.5)	0.059
Type VI complex lesion	114 (38)	29 (39.7)	85 (37.4)	
Type VII calcified lesion	56 (18.7)	12 (16.4)	44 (19.4)	
Type VIII fibrotic lesion	57 (19)	10 (13.7)	47 (20.7)	

Continuous data are shown as the median (interquartile range). Dichotomous data are shown as n (%). The Mann–Whitney test was used for the statistical comparison of continuous variables. Chi-square test was used for categorical variables of more than two classes (ultrasound and histology). US, ultrasound; AHA, American Heart Association.

**Table 3:** Prognostic value of vitamin D deficiency for MACE and all-cause mortality in patients with high-grade carotid artery stenosis undergoing carotid endarterectomy.

	HR per 1-SD	95% CI	*p*-Value
**Univariable**			
MACE	1.5	1.1–2.2	0.026
All-cause mortality	2.2	1.5–3.3	<0.001
**Multivariable model**			
MACE	1.6	1.1–2.6	0.030
All-cause mortality	2.2	1.3–3.6	0.002

MACE was defined as the composite of cardiovascular death, myocardial infarction, transient ischemic attacks, or stroke, as well as atherosclerosis progression in the coronary or peripheral arteries requiring either interventional (percutaneous coronary intervention or peripheral balloon angioplasty with and without stenting) or surgical revascularization (aortocoronary bypass or peripheral bypass). Univariable and multivariable Cox proportional hazard regression models were fit to investigate the predictive value of vitamin D deficiency for MACE and all-cause mortality. The multivariable model was adjusted for age, sex, hypertension, diabetes, coronary and peripheral artery disease, smoking, high-sensitivity CRP, and low-density lipoprotein; HR per 1-SD, hazard ratio per one increase in standard deviation; CI, confidence interval.

## Data Availability

The data supporting the findings of this study are not publicly available due to ongoing data collection and ethical restrictions.

## Supplementary Materials

The following supporting information can be downloaded at: https://www.mdpi.com/article/10.3390/jcm14145163/s1, Figure S1: Study flow chart; Figure S2: Cumulative incidence of major adverse cardiovascular events in symptomatic (panel A) and asymptomatic (panel B) patients with and without vitamin D deficiency. Kaplan-Meier analyses for the cumulative incidence of major adverse cardiovascular events (primary endpoint) in symptomatic (panel A) and asymptomatic (panel B) patients with (blue line) and without (red line) vitamin D deficiency at baseline. Vitamin D levels were assessed as described in the Section 2. *p*-values < 0.05 were considered significant; Figure S3: Kaplan-Meier survival curve in symptomatic (panel A) and asymptomatic (panel B) patients with and without vitamin D deficiency. Kaplan-Meier curves illustrating overall mortality rates. in symptomatic (panel A) and asymptomatic (panel B) patients with (blue line) and without (red line) vitamin D deficiency at baseline. Vitamin D levels were assessed as described in the Section 2. *p*-values < 0.05 were considered significant.
