# Supplementary material for: Vitamin D Deficiency as an Independent Predictor for Plaque Vulnerability and All-Cause Mortality in Patients with High-Grade Carotid Disease
Source: J Clin Med. 2025 Jul 21;14(14):5163. doi: 10.3390/jcm14145163 (PMC12295226; doi:10.3390/jcm14145163)
Supplement: Supplementary file 1 [file jcm-14-05163-s001.zip › jcm-3730660-supplementary.pdf]

Supplemental Figures:

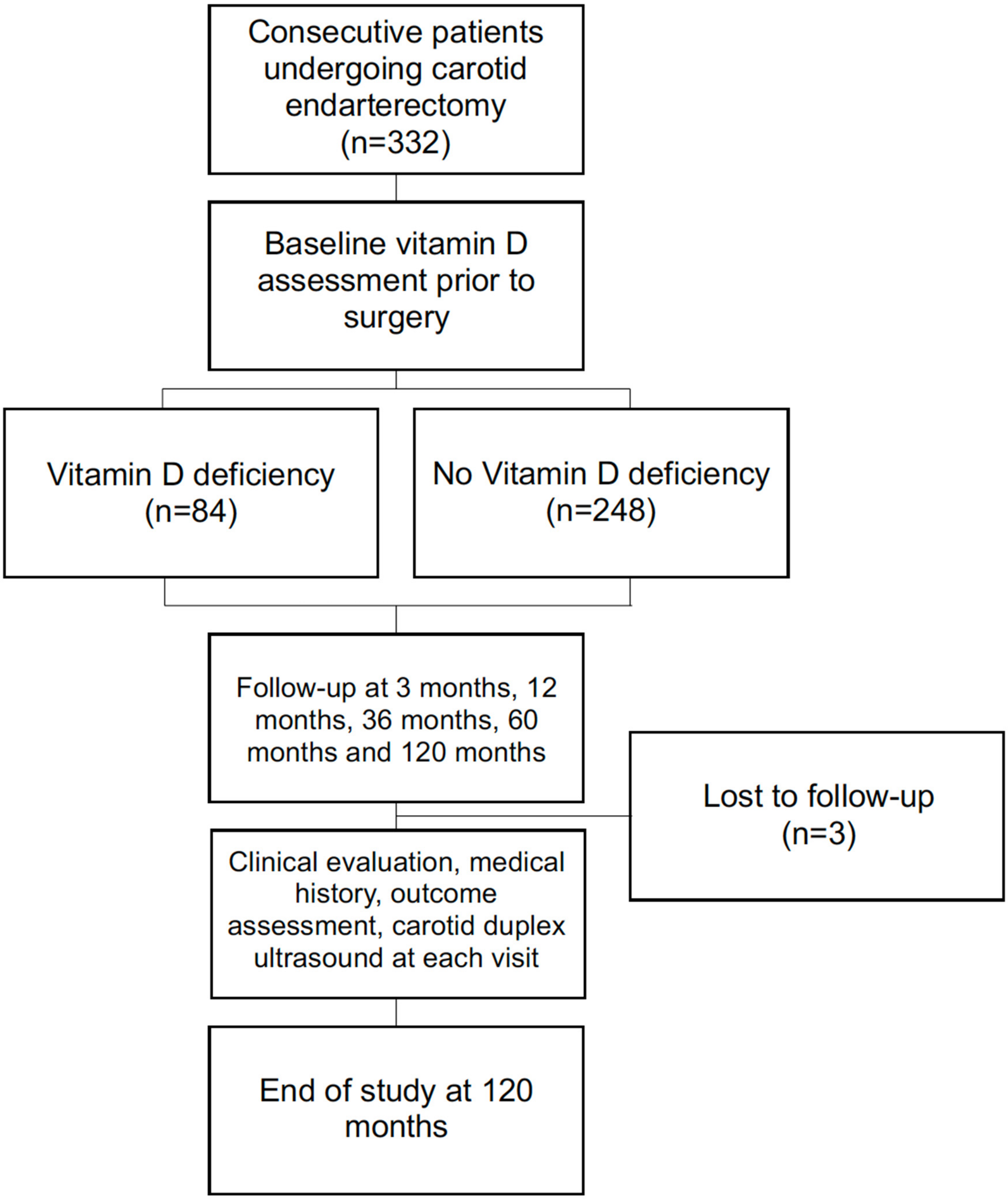

Supplemental Figure S1. Study flow chart.

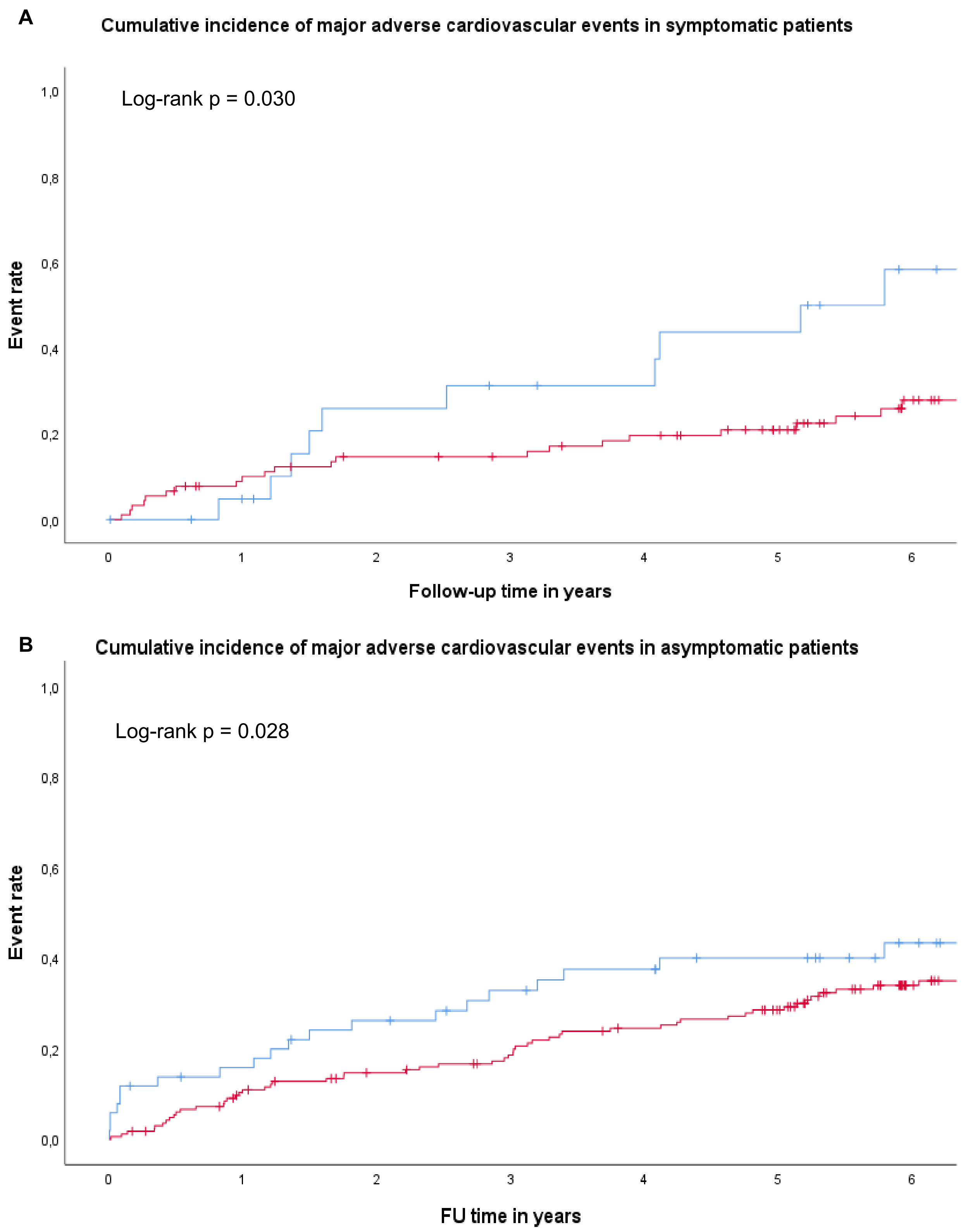

Supplemental Figure S2. *Cumulative incidence of major adverse cardiovascular events in symptomatic (panel A) and asymptomatic (panel B) patients with and without vitamin D deficiency.* Kaplan-Meier analyses for the cumulative incidence of major adverse cardiovascular events (primary endpoint) in symptomatic (panel A) and asymptomatic (panel B) patients with (blue line) and without (red line)

vitamin D deficiency at baseline. Vitamin D levels were assessed as described in the methods section. P-values <0.05 were considered significant.

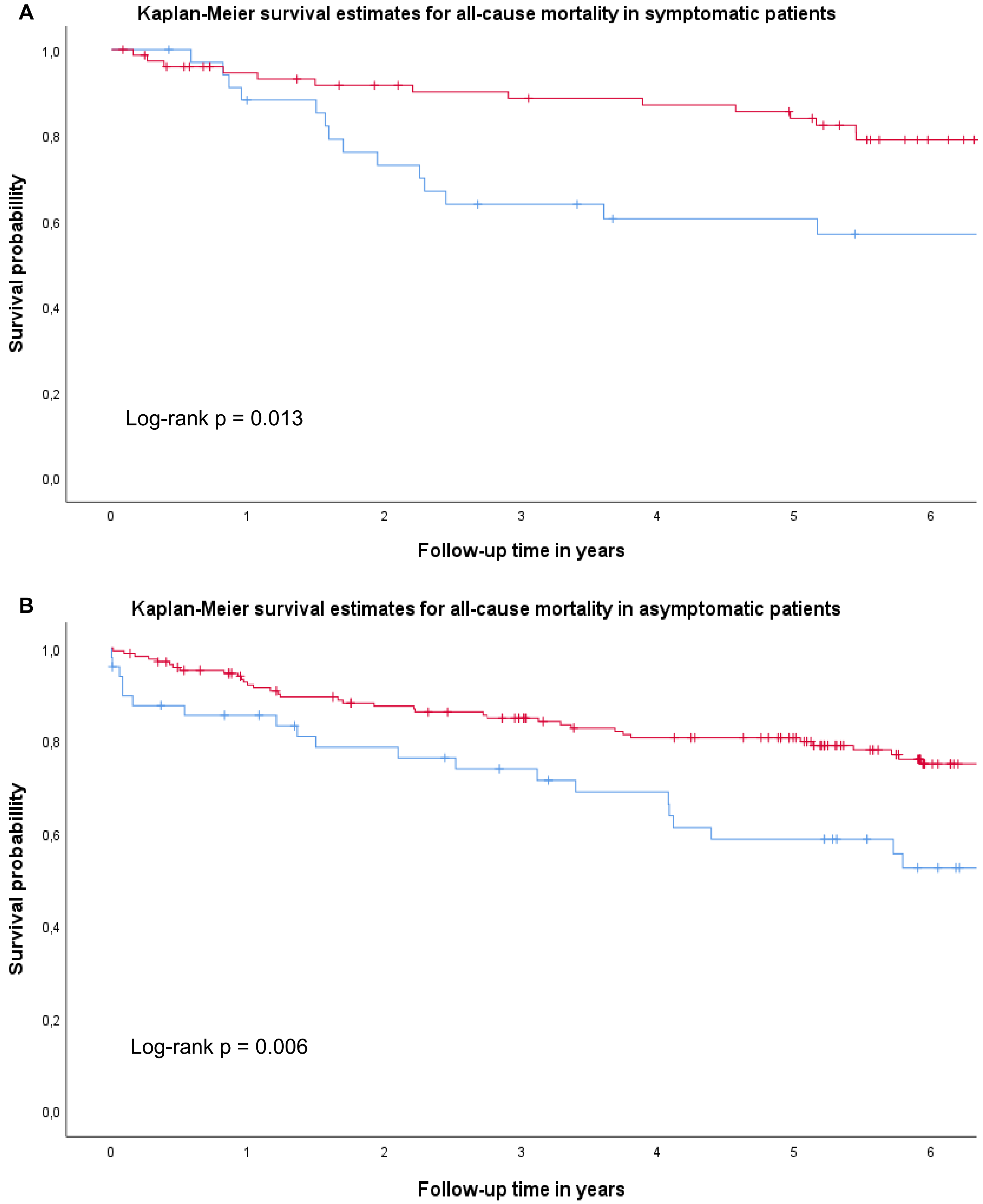

Supplemental Figure S3. *Kaplan-Meier survival curve in symptomatic (panel A) and asymptomatic (panel B) patients with and without vitamin D deficiency.* Kaplan-Meier curves illustrating overall mortality rates. in symptomatic (panel A) and asymptomatic (panel B) patients with (blue line) and without (red line) vitamin D deficiency at baseline. Vitamin D levels were assessed as described in the methods section. P-values <0.05 were considered significant.
